# The Mammalian DM Domain Transcription Factor Dmrta2 Is Required for Early Embryonic Development of the Cerebral Cortex

**DOI:** 10.1371/journal.pone.0046577

**Published:** 2012-10-02

**Authors:** Daijiro Konno, Misato Iwashita, Yoshiaki Satoh, Asuka Momiyama, Takaya Abe, Hiroshi Kiyonari, Fumio Matsuzaki

**Affiliations:** 1 Laboratory for Cell Asymmetry, RIKEN Center for Developmental Biology, Kobe, Hyogo, Japan; 2 Laboratory for Animal Resources and Genetic Engineering, RIKEN Center for Developmental Biology, Kobe, Hyogo, Japan; Universitat Pompeu Fabra, Spain

## Abstract

Development of the mammalian telencephalon is precisely organized by a combination of extracellular signaling events derived from signaling centers and transcription factor networks. Using gene expression profiling of the developing mouse dorsal telencephalon, we found that the DM domain transcription factor Dmrta2 (doublesex and mab-3-related transcription factor a2) is involved in the development of the dorsal telencephalon. Consistent with its medial-high/lateral-low expression pattern in the dorsal telencephalon, *Dmrta2* null mutants demonstrated a dramatic reduction in medial cortical structures such as the cortical hem and the choroid plexus, and a complete loss of the hippocampus. In this mutant, the dorsal telencephalon also showed a remarkable size reduction, in addition to abnormal cell cycle kinetics and defective patterning. In contrast, a conditional *Dmrta2* deletion in the telencephalon, which was accomplished after entry into the neurogenic phase, resulted in only a slight reduction in telencephalon size and normal patterning. We also found that Dmrta2 expression was decreased by a dominant-negative Tcf and was increased by a stabilized β-catenin form. These data suggest that Dmrta2 plays pivotal roles in the early development of the telencephalon *via* the formation of the cortical hem, a source of Wnts, and also in the maintenance of neural progenitors as a downstream of the Wnt pathway.

## Introduction

During the development of the mammalian telencephalon, the regulation of temporal and spatial changes in characteristics of neural progenitors is fundamental for the growth control, regionalization, and layer formation of the cerebral cortex [1]–[3]. Signaling molecules secreted by signaling centers, such as fibroblast growth factors (FGFs) and Wingless-Int (Wnts) molecules, confer regional and temporal specificity to neural progenitors during early cortical development [4]. Intriguingly, these signals also modulate proliferation and differentiation of the neural progenitors in the telencephalon [5]–[8]; for this reason, the control of proliferation and regional specification appear to be tightly linked. Thus, to understand corticogenesis at a molecular level, it is essential to reveal the temporal and spatial regulation of the transcriptional network and its upstream signaling pathways controlled by signaling centers.

The anterior neural ridge (ANR) is a critical signaling center positioned at the anterior midline of the telencephalon. The ANR controls telencephalon formation by modulating rostrocaudal patterning through the secretion of FGFs during the early stages of cortical development [6], [7], [9]–[11]. Furthermore, the cortical hem, another signaling center located in the medial edge of the dorsal telencephalon, regulates mediolateral patterning by the expression of multiple BMPs and Wnts [12], [13] and functions as the organizing center for the development of the hippocampus [5], [14], [15]. Cortical hem-derived Wnt3a signaling regulates the neural progenitor proliferation in the medial part of the dorsal telencephalon [5]. This is presumably accomplished by changing the activity of its downstream nuclear Tcf/Lef effectors from transcriptional repressors to activators through the stabilization of β-catenin.

A number of studies using genetic models have shown that the spatially discrete expression of multiple TFs, including Coup-TFI, Pax6, Emx2, and Sp8, in the developing telencephalon appears to be important for the regional specification and proliferation of neural progenitors [1]–[3]. A remarkable feature of these TFs is that their expression pattern follows a distinct rostrocaudal and/or mediolateral gradient, suggesting that the expression of these molecules is tightly regulated by extracellular signaling from the ANR and cortical hem [1]–[3]. These studies have greatly increased our understanding of molecular mechanisms controlling cerebral cortical development; however, given the complexity of the temporal and regional regulation of corticogenesis, there seems to be several missing links in the TF network regulating cortical development.

In this study, we aimed to identify the molecules expressed in the developing telencephalon in a temporally restricted manner using gene expression profiling of neural progenitors from the dorsal telencephalon. We then focused on the function of the DM domain containing transcription factor Dmrta2 based on its unique expression pattern during cortical development. We thus investigated its role in the development of the dorsal telencephalon using a gene targeting strategy.

## Materials and Methods

### Animals

Embryonic stages were calculated by defining noon on the day of vaginal plug as embryonic day 0.5 (E0.5); the day of birth was defined as postnatal day 0 (P0). All animal manipulations were performed according to the guidelines for animal experiments at the RIKEN Center for Developmental Biology.

### Purification of Neural Progenitors

Neural progenitors were purified using FACS from dorsal telencephalons collected at four different stages of mouse development (E10.5, E12.5, E15.5, E18.5) from pHes1-d2EGFP transgenic mice [16], [17]. These animals were a gift from R. Kageyama (Kyoto University, Kyoto, Japan). Cells were collected directly into TRIzol LS reagent (Invitrogen).

### Microarray Analysis

Total RNA was extracted using the RNeasy MinElute kit (Qiagen, Tokyo, Japan). The cDNA synthesis and cRNA labeling reactions were performed according to the two-cycle protocol provided by Affymetrix. Affymetrix high-density oligonucleotide arrays for *Mus musculus* (Mouse Genome 430 2.0 Array) were hybridized, stained and washed, as described in the expression analysis technical manual (Affymetrix). The expression values were summarized using the RMA method. The resulting expression values were used in all subsequent analyses.

### Plasmids

All mammalian expression vectors were based on pCAG [18]. The expression plasmid for NLS-tagged EGFP, pCAG-EGFP3NLS, was described previously [18]. The expression plasmids for dominant-negative tcf3 and constitutive-active β-Catenin, pCAG-DN-Tcf3 and pCAG-CA-βCat, were gifts from T. Iwano (CDB, RIKEN, Kobe, Japan). To construct the bacterial expression plasmids, cDNAs encoding the C-terminus of Dmrt3 and Dmrta2, as described below, were amplified and cloned into pGEX-6X (GE Healthcare, UK). To construct the expression plasmids for Flag- or HA-tagged Dmrta2, a cDNAs encoding the full-length Dmrta2 without the stop codon were amplified by PCR and then cloned into the pCAG-Flag-N1 plasmid [19]. All plasmids were confirmed using DNA sequencing.

### Generation of Polyclonal and Monoclonal Antibodies

Polyclonal antibodies for Dmrt3 and Dmrta2 were generated by immunizing rabbits with glutathione S-transferase (GST)-fused C-terminal mouse Dmrt3 (aa 147–476) and mouse Dmrta2 (aa 129–531). The antisera were preabsorbed with GST protein, followed by purification using antigen coupled to a Sepharose 4B gel matrix.

A rat monoclonal antibody that specifically recognized Dmrta2 was generated based on the rat lymph node method [20]. A WKY/izm rat was immunized with GST-fused recombinant Dmrta2. After three weeks, rat lymphocytes were fused with mouse myeloma SP2/W cells. Hybridoma clones producing Dmrta2-specific antibody were screened by ELISA and immunostaining. Finally, a hybridoma clone producing a monoclonal antibody (1D1) was selected.

Antibody specificity was confirmed by Western blotting (Figure S1).

### Mutant Mice

To generate conditional mutants for *Dmrta2*, we constructed a targeting vector harboring the loxP-flanked exon 2 of *Dmrta2* and a Frt-flanked neomycin resistant cassette (neo-cassette). The targeting vector was introduced into the TT2-derived mouse ES cells by electroporation as described previously [21]. After isolation of three homologous recombinants in the ES cells, the clones were introduced into 8-cell embryos of CD1 mice. To delete the neo-cassette, chimeric mice were mated with *ACTB-FLPe* mice (Stock No. 003800, The Jackson Laboratory, Maine, USA), which ubiquitously express Cre recombinase under the control of the human *ACTB* promoter. Two independent mutant mouse lines were analyzed to confirm the phenotype (Accession No. CDB1049K: http://www.cdb.riken.jp/arg/mutant%20mice%20list.html). For experiments using *Dmrta2* null mutants, we crossed *Dmrta2* conditional mutants to *EIIa-Cre* transgenic mice (Stock No. 003724, The Jackson Laboratory). For the conditional deletion of *Dmrta2* in the central nervous system, we crossed *Dmrta2* conditional mutants with *Nestin-Cre* transgenic mice [22] and analyzed the phenotype of heterozygotic and homozygotic *Dmrta2* mutants containing the hemizygous *Nestin-Cre* transgene. All mutant phenotypes were analyzed on a C57BL/6 mouse background. The genotypes of the resulting mutant mice were determined according to the size of their PCR products (wild-type: 319 bp, floxed: 516, deleted: 200 bp) using the described primers (Primer-1: GATCCTAGTGAACCTCTTCGAGGGAC, Primer-2: GAGTTGCAATTGCTGTACGGCACTG, Primer-3: GAGCCACAGTTAAGTAGTTGGAG).

*Dmrta2*-heterozygous mice exhibited normal fertility and CNS development, and we therefore used the heterozygous mice as a control in this study.

### *In situ* Hybridization

Section and whole-mount *in situ* hybridization using digoxigenin-labeled cRNA antisense probes were performed according to standard methods. The cDNA sequences of *Dmrt3* (937–1679 nt, accession number BC052041), *Dmrta1* (603–1585 nt, accession number NM_175647), *Dmrta2* (1015–1807 nt, accession number NM_172296), *Wnt3a* (546–1104 nt, accession number NM_009522), and *Transthyretin (Ttr)* (471–978 nt, accession number AK014454) were amplified by PCR primers containing the bacteriophage T7 RNA polymerase promoter sequence (5′-GCGGTAATACGACTCACTATAGGGC-3′) to prepare the cRNA probes.

### Histology

Hematoxylin and Eosin (HE) staining was performed according to standard methods. Briefly, Mayer’s Hematoxylin solution and 1% Eosin Y solution (Wako Pure Chemical Industries, Osaka, Japan) were sequentially used for nuclear and cytoplasmic staining, respectively.

Immunofluorescence staining was performed, as described previously [18]with minor modifications. Briefly, mouse brains were fixed in 1% paraformaldehyde in 0.1 M phosphate buffer (pH 7.4) at 4°C for 2 h. The primary antibodies used were as follows: Sox2 (Y-17; Santa Cruz Biotechnology, Santa Cruz, CA, USA), Map2 (clone HM-2; Sigma-Aldrich, Tokyo, Japan), Otx2 (Abcam, Tokyo, Japan), Otx2 (R&D systems, MN, USA), Reelin (clone CR-50; MBL, Nagoya, Japan), Tbr1 (a gift from Dr. Rovert Hevner, University of Washington, WA), Satb2 (clone SATBA4B10; Abcam), βIII-tubulin (clone Tuj1; Covance, Princeton, NJ, USA), phosphorylated histone H3 (clone 6G3; Cell Signaling, Danvers, MA), Cre recombinase (clone 2D8; Millipore, Billerica, MA, USA), Prox1 (R&D systems), Pou3f2 (Brn-2) (C-20; Santa Cruz Biotechnology), and Ki67 (SP6; Abcam), Pax6 (Covance). The primary antibodies were detected with the secondary antibodies conjugated to Alexa488, Cy3, and Cy5 (Jackson, West Grove, PA, USA). For staining with mouse monoclonal antibodies, sections were blocked with monovalent Fab fragments (Jackson) to reduce the background signals on the mouse tissues. Apoptotic cells were detected by TUNEL (terminal deoxynucleotidyl transferase dUTP nick-end labeling) staining using In Situ Cell Death Detection kit (Roche Diagnostics Japan, Tokyo, Japan). All fluorescent images were acquired by Olympus FV1000 confocal microscope (Olympus, Tokyo, Japan).

### DNA Labeling with EdU

Pregnant mice were injected intraperitoneally with 100 µl of EdU (10 mg/ml) (Invitrogen, Tokyo, Japan). Animals were euthanized at 30 min after injection to visualize the S-phase cells. The cell cycle exit rate was estimated as the ratio of Ki67-negative/EdU-positive cells to all EdU-positive cells at E12.5 (18 h after EdU injection) or E15.5 (24 h after EdU injection).

### *In utero* Electroporation

*In utero* electroporation was performed at E11.5, as described previously (Saito et al., 2001). Briefly, CD1 mouse embryos were electroporated with expression plasmids using an electroporator (CUY21, NEPPAGENE, Tokyo, Japan) at the following concentrations: pCAG-EGFP3NLS (0.5 µg/µl), pCAG-DN-Tcf3 (1.0 µg/µl), and pCAG-CA-β-catenin (1.0 µg/µl). Electroporated brains were analyzed 24 h after electroporation by immunofluorescence.

### Quantifications

To quantify the expression levels of Dmrt3 and Dmrta2, the immunostaining fluorescence intensity was evaluated using Olympus FV10-ASW v1.7 software. All statistical analyses were performed with the Student’s *t*-test using Prism 5 or Excel software. The data were expressed as the mean±SD.

## Results

### Identification of Young Neural Progenitor-enriched Genes

**Figure 1:**
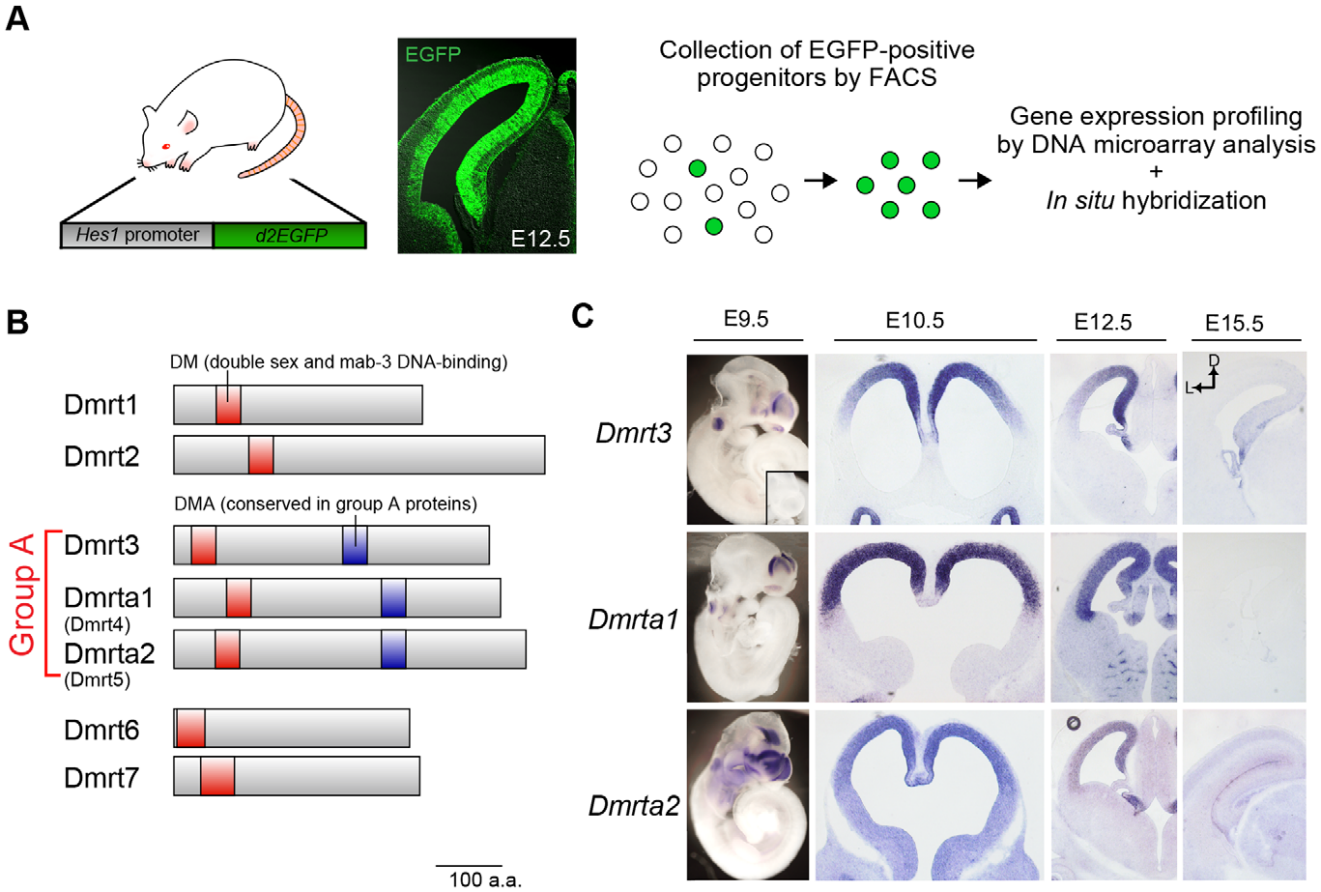
Group A subfamily of *Dmrt* genes are expressed in a temporally and spatially specific manner in the developing telencephalon. (**A**) Strategy for the gene expression profiling of neural progenitors isolated from the dorsal telencephalon of pHes1-d2EGFP mice at different developmental stages (E10.5, E12.5, E15.5, and E18.5). Fluorescence images show EGFP expression in the telencephalon of pHes1-d2EGFP mice at E12.5. Note the specific expression of EGFP in the neuroepithelium. (**B**) The *Dmrt* gene family consists of seven DM-domain proteins. Dmrt proteins share highly conserved protein motifs, including the DM (double sex and mab-3 DNA binding) domain and are subdivided into a few sub-groups by their sequence homology. *Dmrt3*, *Dmrta1 (Dmrt4)*, and *Dmrta2 (Dmrt5)* are categorized as group A because their products share a highly conserved protein domain DMA. (**C**) *In situ* hybridization reveals that group A Dmrt family genes are enriched in the dorsal region of the telencephalon. Expression of these genes is detected in the dorsal telencephalon at E9.5 and dramatically reduced at E15.5. Robust expression is also observed in the otic vesicle at E9.5 and in the olfactory epithelia in all stages examined.

To identify the genes predominantly expressed in the neural progenitors of the dorsal telencephalon during the early stages of cortical development, we first used FACS to isolate progenitor cells from the dorsal telencephalon of Hes1-reporter mice at four developmental stages: the proliferative stage (E10.5), the early neurogenic stage (E12.5) and the later neurogenic stages (E15.5 and E18.5) (Figure 1A). Hes1-reporter mice were shown to express destabilized EGFP in their neural progenitors under the control of the Hes1 promoter (pHes1-d2EGFP) [16], [17]. Thus, the neural progenitors were easily purified using this method, with low levels of contamination from other cell types.

The gene expression profiles of the purified neural progenitors analyzed with DNA microarray demonstrated a steady expression level of *Sox2*, a neural progenitor marker, at all examined stages, as expected (Table S1). These data indicate that a pure population of neural progenitors was efficiently isolated using our strategy. We next found that 483 probe sets were abundantly expressed in the brain at E10.5 and E12.5, and that their expression gradually decreased in the later stages (Table S1 and data not shown).

Of these genes, we were most interested in the expression of three related genes, *Dmrt3*, *Dmrta1 (Dmrt4)*, and *Dmrta2 (Dmrt5)*, because of their putative functions as DNA-binding factors (Figure 1B) and their unique expression pattern in the developing telencephalon. *In situ* hybridization revealed that these genes were expressed as early as E9.5 and were robustly expressed in the dorsal telencephalon at E10.5 and E12.5 (Figure 1C). We found that *Dmrt3* and *Dmrta2* had medial-high to lateral-low gradient patterns of expression, while *Dmrta1* did not show a graded pattern of expression (Figure 1C). Taken together, these data suggest that *Dmrt3* and *Dmrta2* are involved in the spatiotemporal pattern of specification and/or in the differentiation of telencephalic neural progenitors.

### Dmrt3 and Dmrta2 are Robustly Expressed in the Medial Telencephalon

**Figure 2:**
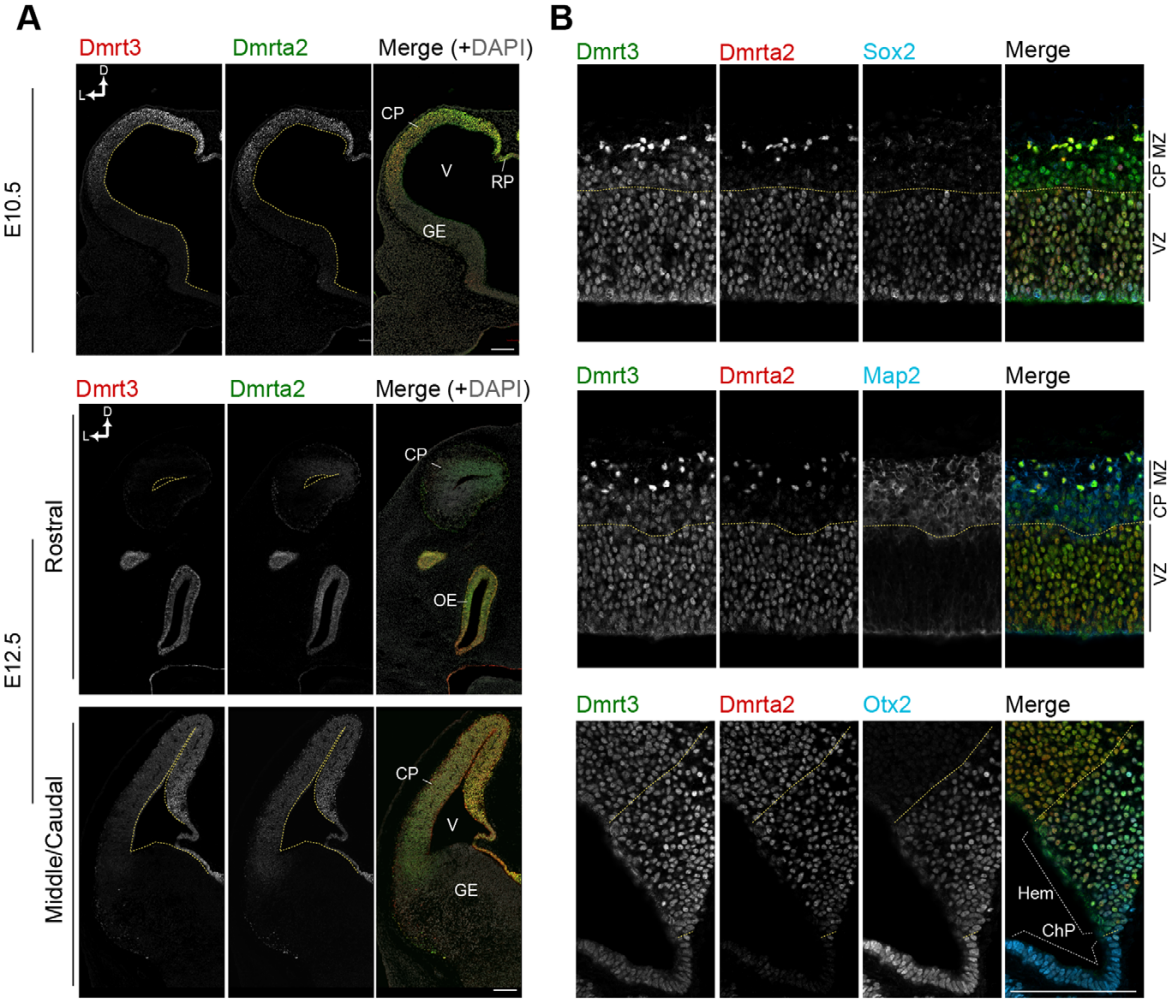
Expression of Dmrt3 and Dmrta2 in the developing telencephalon. (**A**) Immunostaining for Dmrt3 and Dmrta2 with DAPI counterstaining on coronal sections of the telencephalon at E10.5 and E12.5. (**B**) Immunostaining for Sox2 (top), Map2 (middle), and Otx2 (bottom) with Dmrt3 and Dmrta2 on coronal sections of the telencephalon at E13.5. Images show the magnified view of the dorsolateral area of the telencephalon. Dotted lines indicate the ventricular surface of the telencephalon (A), the border of the ventricular zone and cortical plate (B, top and middle), and the border of the hippocampal primordium and cortical hem (B, bottom). CP, cortical plate; OE, olfactory epithelium; V, ventricle; RP, roof plate; GE, ganglionic eminence; D, dorsal; L, lateral. ChP, choroid plexus; Hem, cortical hem. Scale bar, 100 µm.

We next investigated the expression of Dmrt proteins by immunostaining with specific antibodies (Figure S1). Consistent with our *in situ* hybridization analysis, immunofluorescence staining for Dmrt3 and Dmrta2 revealed a graded expression pattern in the dorsal telencephalon at E10.5 and E12.5 (Figure 2A). Both Dmrt3 and Dmrta2 proteins were detected in all Sox2-positive neural progenitors in the midial high-lateral low manner in the dorsal telencephalon (Figure 2B, top). Dmrt3 was also detected in Map2-positive cortical plate neurons, although its expression level was much lower in neurons than in neural progenitors (Figure 2B, middle). In the medial cortex, Dmrt3 was strongly expressed in the cortical hem and its derivative, the choroid plexus, both of which are marked by Otx2 expression [23] (Figure 2B, bottom). Dmrta2 was also strongly expressed in the cortical hem while its expression in the choroid plexus was faint.

### Reduction of the Telencephalon in *Dmrta2* Mutant Embryos

To examine the function of Dmrt3 and Dmrta2 *in vivo*, we first generated *Dmrt3*-knockout mice using KOMP (Knockout Mouse Project)-derived Dmrt3-knockout ES cells. *Dmrt3* mutants showed a 16% decrease in the size of the neocortical area at postnatal day 0 (P0) compared with that of control brains (data not shown). However, we could not find abnormalities of proliferation and differentiation in neocortical neural progenitors at embryonic stages. We then focused on the function of Dmrta2 using *Dmrta2*-knockout mice. *Dmrta2*-mutant mice were generated by flanking *Dmrta2* exon 2, which includes the DM domain critical for DNA-binding, with loxP sequences (Figure S2). LoxP-flanked (floxed)-*Dmrta2* mutants (*Dmrta2^flox/flox^*) were crossed with an *EIIa-cre* transgenic mouse line, which expresses Cre recombinase ubiquitously, to generate *Dmrta2*-null mutants (*Dmrta2^−/−^*).

**Figure 3:**
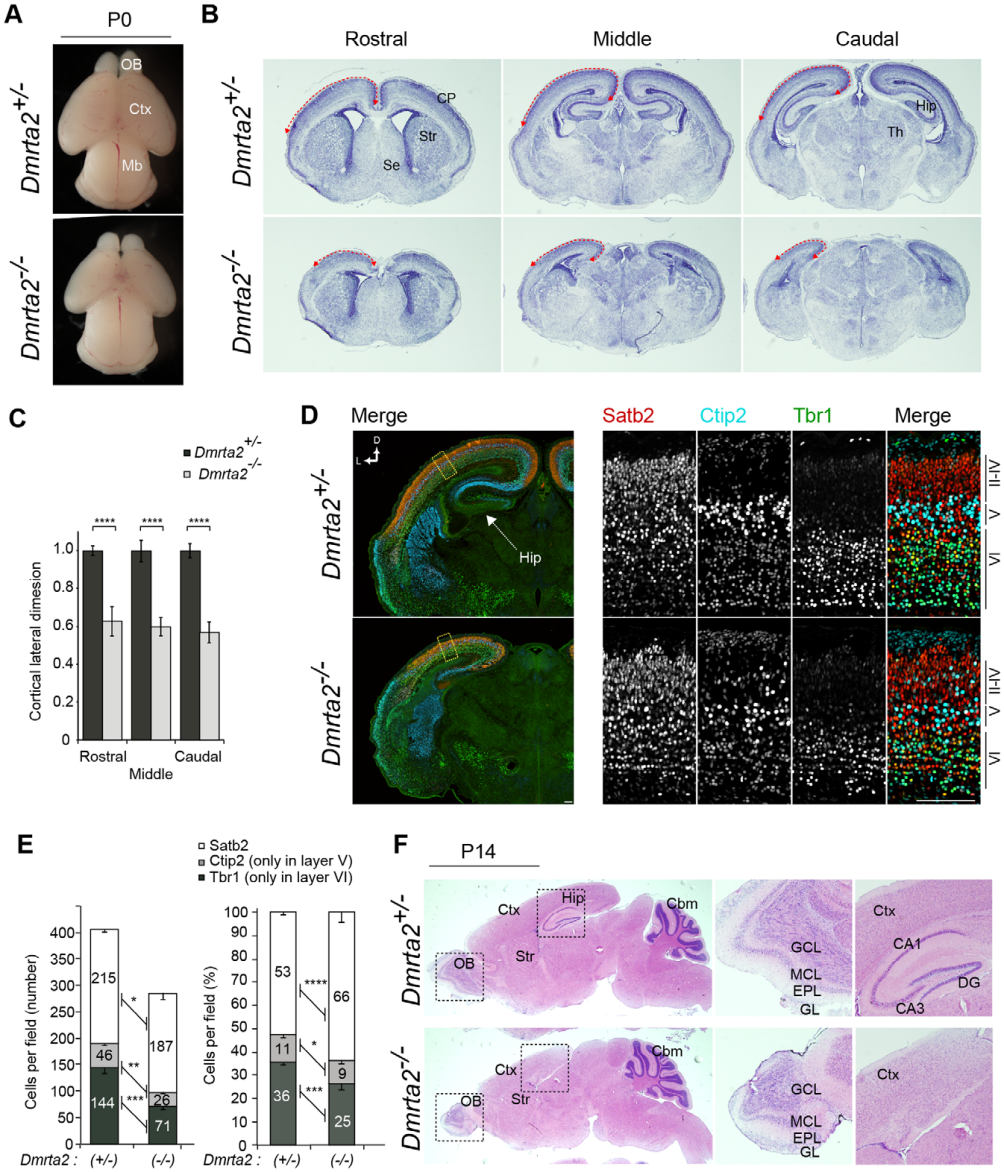
Reduced cortical size in *Dmrta2*-deficient mice. (**A**) Newborn (P0) brains of *Dmrta2* mutants. (**B**) HE staining of 25 µm coronal sections of P0 *Dmrta2* mutant brains at the equivalent plane of the rostral, middle, and caudal regions. (**C**) The relative length of the cortical lateral dimension (arrows in B) in *Dmrta2* mutants. The length is expressed as a relative value to the mean of the data from *Dmrta2^+/−^* control brains. *n* = 3. ****p<0.0001. (**D**) Immunostaining for Satb2, Ctip2, and Tbr1 in the dorsolateral area of the cerebral cortex at P0. Scale bar, 100 µm. (**E**) Quantification of cortical layer marker-positive cells in a 100-µm-wide segment of the cortical wall as shown in (D). Data are expressed as the number (left) or a percentage (right) of a total of each marker-positive cells per field. *n* = 3. *p<0.05, **p<0.01, ***p<0.001, ****p<0.0001. (**F**) HE staining of 25 µm sagittal sections of mutant brains at P14. Middle and right panels show higher magnifications of the boxed regions of the hippocampus and the olfactory bulb in the right panels. OB, olfactory bulb; Mb, midbrain; Ctx, cerebral cortex; Str, striatum; Se, septum; Hip, hippocampus; EPL, external plexiform layer; GCL, granule cell layer; GL, glomerular layer; MCL, mitral cell layer; DG, dentate gyrus.

To examine the effect of *Dmrta2* deletion on the telencephalon, we analyzed the size and gross morphology of the brain at P0. In *Dmrta2^−/−^* mice, the size of the telencephalon appeared to decrease compared to that of the control brains, although the midbrain showed no changes in either morphology or size (Figure 3A). Consistent with this observation, the size of the cerebral cortical area defined by HE staining in *Dmrta2^−/−^* mutants was reduced by approximately 40% compared with the control brains, as assessed in three different planes of the sections (Figure 3B, C). Most evidently, the hippocampal structure almost disappeared in *Dmrta2^−/−^* mutants, and the formation of the entire medial structure was disorganized (Figure 3B, D, E, F). The size of the olfactory bulb was also reduced in the mutant, while the layer formation of the major olfactory bulb appeared to be normal (Figure 3F). Immunostaining for Reelin, which is a marker for mitral cells of the olfactory bulb in adulthood, confirmed that these cells were correctly organized in a single layer (data not shown). To more closely examine the neocortical development of *Dmrta2* mutants, we performed immunofluorescence staining on P0 coronal brain sections for Tbr1, which is strongly expressed in the cortical layer VI and in the subplate neurons [24], Ctip2, which is highly expressed in a subset of corticospinal motor neurons [25], and Satb2, which is predominantly expressed in the callosal projection neurons [26], [27]. *Dmrta2^−/−^* mice were found to have correct positioning of their Tbr1-, Ctip2-, and Satb2-positive cortical neurons in an inside-out manner, similar to that of the control brains (Figure 3D). We note that the loss of *Dmrta2* slightly affected the positioning of neurons as observed for Ctip2-positive neurons in the layer II-IV (Figure 3D). This might be due to a massive loss of Cajal-Retzius cells, which play important roles in radial migration of cortical neurons, in the mutant telencephalon (Figure S3E). In contrast to the nearly normal layering of cortical neurons, the number of such neurons in *Dmrta2^−/−^* brains appeared to be changed {215.3±4.9 (hetero) vs. 187.7±12.5 (homo) for Satb2, 45.7±4.0 (hetero) vs. 26.0±2.0 (homo) for Ctip2, and 144.3±11.8 (hetero) vs. 71.0±6.1 (homo) for Tbr1} (Fig. 3E). Remarkably, the number and density of the early born neurons per unit area of neocortex were significantly decreased in *Dmrta2^−/−^* brains. In contrast, the number of Satb2-positive late-born neurons was only slightly decreased while their density in the cortical layer increased (Figure 3E). The production of cortical layer specific neurons is tightly controlled by temporal identities and/or cell cycle kinetics in neocortical neural progenitors, which in turn depends on the developmental stages of the brain [28]. For this reason, our observations suggest that Dmrta2 predominantly functions to maintain the early stage-specific features of the progenitors during cerebral cortical development. This finding is consistent with the observed temporal pattern of Dmrta2 expression. Next, we investigated the details of Dmrta2 function in the early embryonic development of the cerebral cortex.

### Severe Disorganization of the Medial Cortex in *Dmrta2* Mutants

One of the early critical steps during telencephalic development is the formation of signaling centers, such as the cortical hem and the anterior neural ridge [4].

**Figure 4:**
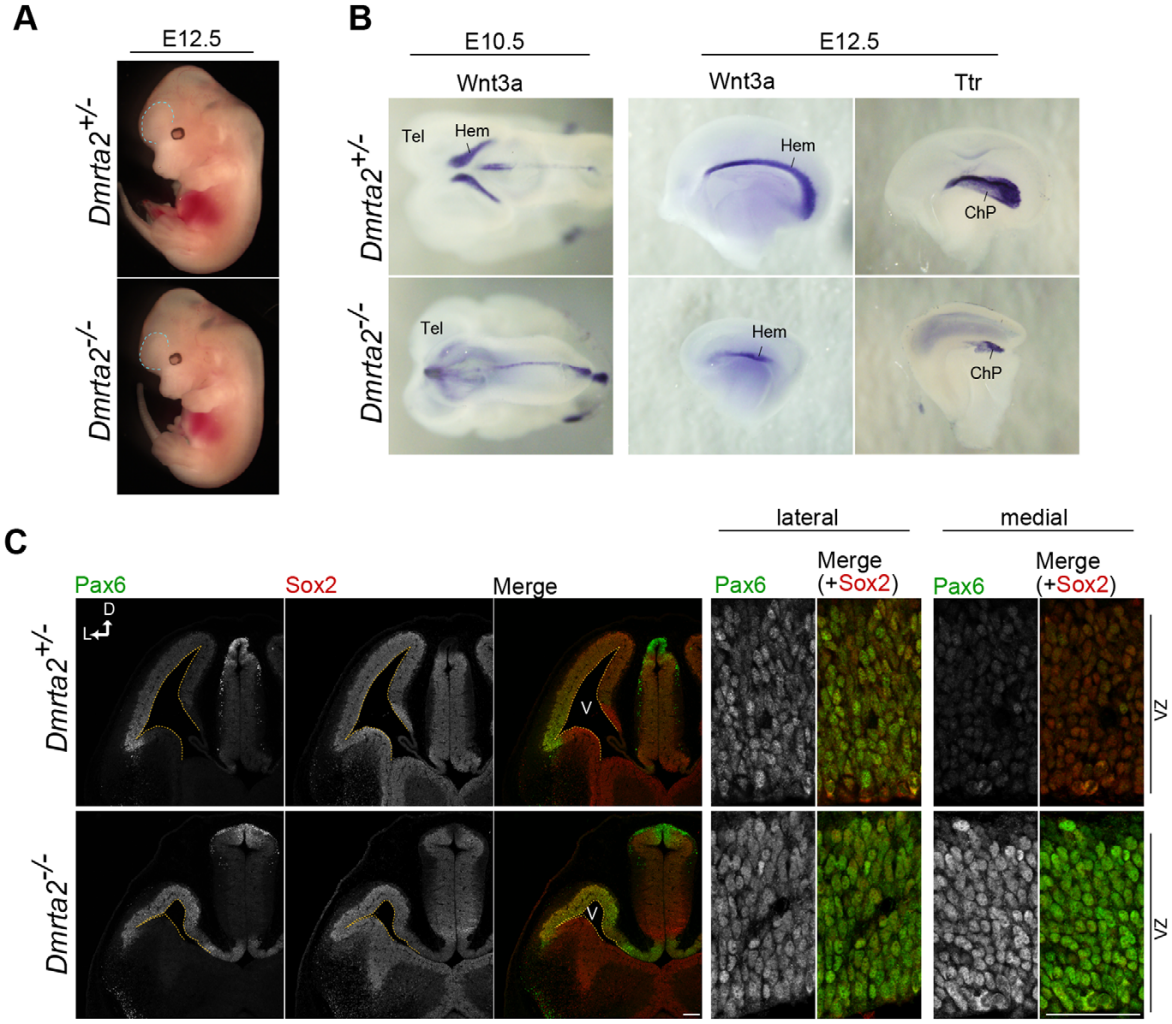
Impairment of cortical hem formation and cell cycle progression in *Dmrta2^−/−^* embryos. (**A**) Gross morphology of embryos from E12.5 *Dmrta2* mutant embryos. (**B**) *In situ* hybridization for *Wnt3a* of E10.5 brains as viewed from the top (left), and for *Wnt3a* and *Transthyretin (Ttr)* of E12.5 cerebral hemispheres as viewed from the medial surface (middle and right). (**C**) Immunofluorescence for Pax6 and Sox2 at E12.5. Right panels show higher magnifications of the lateral and medial neuroepithelial regions of the left panels. Scale bar, 50 µm.

The nearly complete loss of the hippocampus in *Dmrta2^−/−^* brains raises the possibility that Dmrta2 is involved in the formation of the cortical hem, a signaling center located in the medial edge of the telencephalon. Indeed, the cortical hem-derived signal is known to regulate the growth of the hippocampal primordium [5]. Therefore, we investigated whether *Dmrta2* deletion affects the formation of the hem at E10.5 and E12.5 by *in situ* hybridization for *Wnt3a*, a marker for the cortical hem. Although we did not observe any changes in body size and gross morphology in *Dmrta2^−/−^* embryos (Figure 4A), *Wnt3a-*positive cortical hem region was dramatically reduced by E10.5, and only small portion was detected at E12.5 (Figure 4B). Consistent with a previous report describing the involvement of the cortical hem in choroid plexus formation [14], [29], the expression of *transthyretin (Ttr)*, a marker for the choroid plexus, was also significantly reduced in *Dmrta2^−/−^* mutants (Figure 4B). To examine the role of Dmrta2 in the formation of signaling centers other than the cortical hem, we checked the expression of *Fgf8*, a mediator of brain patterning expressed in the anterior midline of the telencephalon, but found no change in its expression (data not shown). We further confirmed the sustained disorganization of the medial structures of the telencephalon by immunostaining for regional markers; the expression of Otx2, which is predominantly expressed in the cortical hem and choroid plexus, was observed only in a very small population of cells compared to the control brain (Figure S3). Prox1, which is expressed in the hippocampal primordium under the control of the Wnt signaling during embryonic brain development [30], almost disappeared in *Dmrta2^−/−^* mutants at E15.5 (Figure S3). These data strongly suggest that cortical hem function is severely compromised in *Dmrta2^−/−^* mutants. In addition, *Dmrta2^−/−^* brains lost their lateral-high to medial-low gradient of Pax6 expression, which plays a critical role in cell fate determination and cell cycle progression in the mammalian CNS (Figure 4C). Control brains showed an expression of Pax6 in the dorsal telencephalon with the gradient, whereas *Dmrta2^−/−^* mutant brains showed a uniform expression of Pax6 expression in the entire dorsal neuroepithelium (Figure 4C). Taken together, these observations indicate that the genetic ablation of *Dmrta2* causes the hypoplasia or loss of the medial structures in the telencephalic cortex, thus implying that Dmrta2 plays a pivotal role in the regulation of neocortical patterning and in the formation of the signaling center.

### Altered Cell Cycle Kinetics in *Dmrta2* Mutants

**Figure 5:**
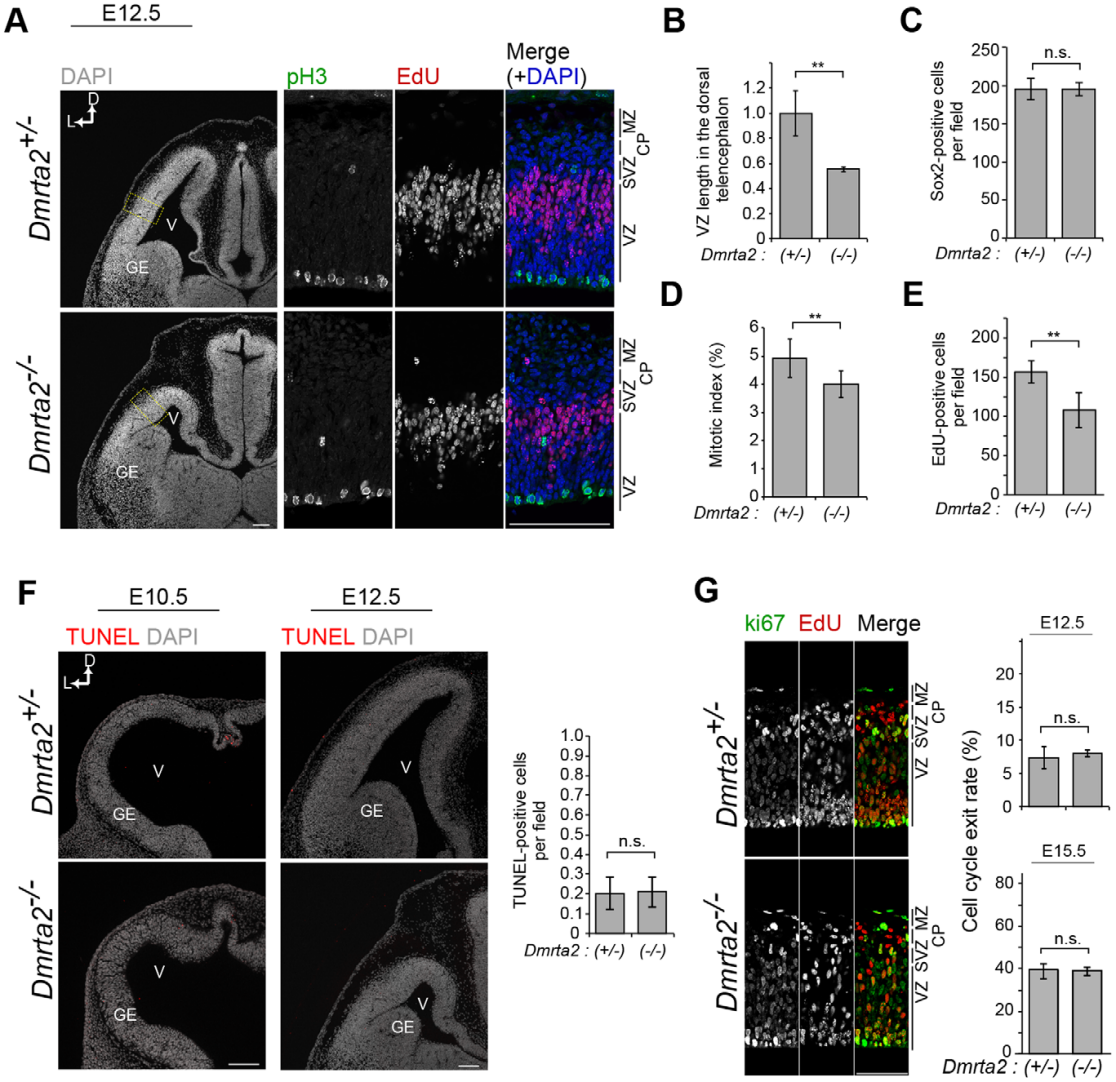
Altered cell cycle kinetics in *Dmrta2^−/−^* embryos. (**A**) DAPI staining (left) and immunostaining (right) for phosphorylated histone H3 (pH 3) and EdU (pulse-labeled for 30 min) on coronal sections of the telencephalon at E12.5. Right panels show higher magnifications of boxed regions with staining images. (**B**) The length of the ventricular surface in the dorsal telencephalon at E12.5. The value is represented relative to the control value. *n* = 4. **p<0.01. (**C**) The number of Sox2-positive cells in a 100-µm-wide segment at E12.5. *n* = 4. (**D**) The mitotic index is represented as the percentage of pH3-positive cells located at the apical side of the VZ among the Sox2-positive cells in the dorsolateral regions of the telencephalon at E12.5. *n* = 9 **p<0.01. (**E**) The number of EdU-positive cells in a 100 µm-wide segment in *Dmrta2* mutants at E12.5. *n* = 4 **p<0.01. (**F**) Fluorescence images of TUNEL staining with DAPI staining at E10.5 and E12.5. Quantification of TUNEL-positive cells is expressed as the number in a 100 µm-wide segment. (**G**) Double immunofluorescence for Ki67 and EdU (pulse labeled for 24 h) in mutant brains to analyze the cell cycle exit rate at E12.5. The rate is expressed as the percentage of Ki67(−)EdU(+) cells of the total EdU-positive cells. ns, not significant. Scale bar, 100 µm.

As shown above, *Dmrta2* mutants showed severe defects in the formation of the medial cortex in addition to dramatic changes in the size of the telencephalon at P0 and P14 (Figure 3). These results raise the possibility that the cell cycle kinetics of neural progenitors is altered in *Dmrta2^−/−^* mutants, such that a smaller number of neural progenitors is formed or maintained during early cortical development. Consistent with this, *Dmrta2^−/−^* mutants showed decreased size of the telencephalon at E12.5 (Figure 5A). The number of progenitor cells was smaller in the dorsal telencephalon of *Dmrta2^−/−^* mutants than that of controls based on the dimension of the dorsal telencephalon (the length of the ventricular zone) (Figure 5B) and the density of Sox2-positive progenitors (the number of these cells in a unit area) (Figure 5C). This difference in the number of the progenitors appeared to occur between E10.5 and E12.5 because no obvious difference was detected in the size of the telencephalon between *Dmrta2* homozygotes and heterozygotes at E10.5 (data not shown). We next examined the cell cycle kinetics at E12.5 by checking the mitotic index, which we defined as the percentage of phosphorylated histone H3 (pH3)-positive cells in the Sox2-positive progenitors, and the number of S-phase cells in the dorsolateral area of the mutant brains. We found that the mitotic index was slightly but significantly decreased in *Dmrta2^−/−^* brains {4.91±0.67 (hetero) vs. 4.00±0.47 (homo)} (Figure 5D). The number of S-phase cells, labeled by 30 min *in vivo* incorporation of EdU, also decreased by 68.5% compared to the control (Figure 5E). We also examined the number of apoptotic cells and the cell cycle exit rate, and we found that the number of TUNEL-positive apoptotic cells and the cell cycle exit rate were not significantly different between control and mutant tissues (Figure 5F, G, and data not shown), suggesting that the defective increase in the progenitor population in *Dmrta2* mutants was unlikely to originate from the apoptosis of progenitors or from premature neurogenesis (see discussion). Taken together, these data suggest that alteration of cell cycle kinetics in progenitors during early brain development is at least partly involved in the dramatic size reduction observed in the dorsal telencephalon of *Dmrta2^−/−^* brains.

### Conditional Ablation of *Dmrta2* in Neurogenic Stages Causes a Moderate Reduction in Brain Size

**Figure 6:**
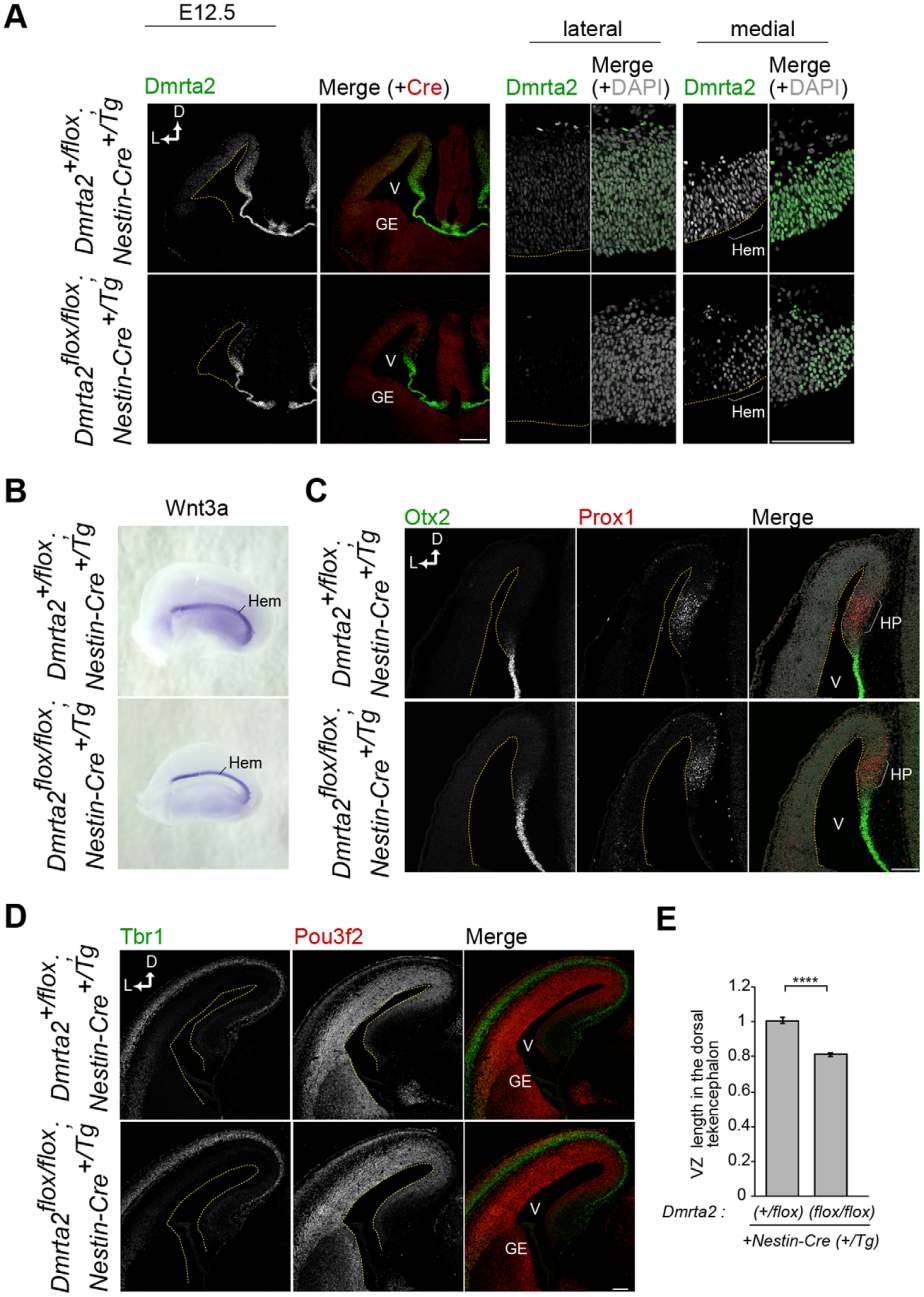
Conditional ablation of *Dmrta2* from neurogenic stages causes a moderate reduction in brains size with no severe patterning defects. (**A**) Double immunofluorescence for Dmrta2 and Cre recombinase at E12.5. Right panels show higher magnifications of the lateral and medial regions shown in the left panels. (**B**) *In situ* hybridization for *Wnt3a* of E12.5 cerebral hemispheres as viewed from the medial surface. (**C**) Double immunofluorescence for Otx2 and Prox1 at E12.5. HP, hippocampal primordium. (**D**) Double immunofluorescence for Tbr1 and Pou3f2. (**E**) Quantification of the ventricular surface dimension in the dorsal telencephalon of *Dmrta2* mutants at E15.5. The length is expressed as a relative value compared to the mean of the data from *Dmrta2^+/flox^*;*Nestin-Cre^+/TG^* control brains. *n* = 4. ****p<0.0001. Dashed lines in (A), (C), and (D) indicate the ventricular surface. Scale bar, 100 µm. HP, hippocampal primordium.

To further clarify the predominant role of Dmrta2 in the early development of the cerebral cortex, we conditionally ablated *Dmrta2* using the *Nestin-Cre* transgenic line. This mouse line is useful to investigate the function of genes after the initiation of the neurogenic phase of brain development because Cre recombinase in this line is not fully active in the dorsal telencephalon until E12.5 [22], [31]. In *Dmrta2-*conditional mutants (cKO) at E12.5, Dmrta2 expression was nearly undetectable in the lateral cortex. However, many cells continued to express Dmrta2 at relatively low levels compared to those of Dmrta2 expression in the medial cortex of the control brains. This suggests that Dmrta2 ablation is completed around E12.5 (Figure 6A). *Dmrta2*-cKO brains exhibited normal formation of the cortical hem (Figure 6B) and the hippocampal primordium in the medial cortex (Figure 6C), indicating that development of the cortical hem and the subsequent specification of the hippocampal primordium were normal. Consistent with the correct formation of the medial structures, the layering of cortical neurons was normal as revealed by immunostaining for Pou3f2 (Brn2), which is predominantly expressed in the upper layer neurons, and Tbr1 at E15.5 (Figure 6D). In contrast to the significant size reduction observed in *Dmrta2^−/−^* telencephalon, no size reduction was observed at E12.5, and only a slight reduction of the ventricular surface dimension was observed at E15.5 in brains with the conditional ablation of *Dmrta2* (Figure 6E, data not shown). We also examined the cell cycle exit rate in *Dmrta2*-cKO brains, however, no significant change was observed (data not shown). Taken together, these results confirm that Dmrta2 predominantly contributes to early events of telencephalic development, including the formation of the cortical hem.

### Canonical Wnt Signaling Regulates the Expression of Dmrt3 and Dmrta2

**Figure 7:**
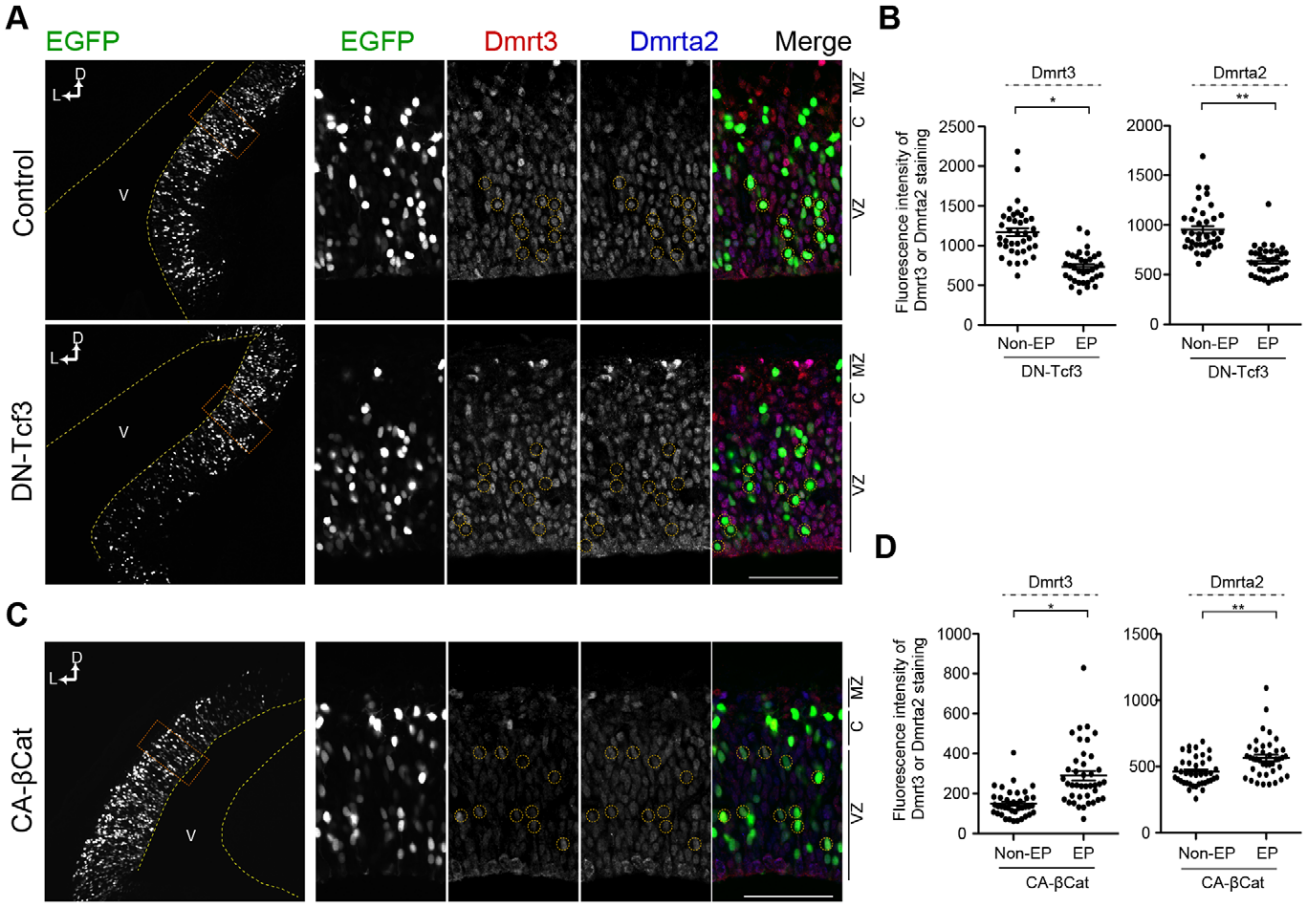
Canonical Wnt signaling regulates Dmrt3 and Dmrta2 expression in the telencephalon. Immunofluorescence staining of Dmrt3 and Dmrta2 with EGFP-fluorescence in the dorsal telencephalon at E13.5. (**A**) Overexpression of a dominant-negative Tcf3 (DN-Tcf3) in the medial cortex at E12.5. (**C**) Overexpression of a constitutive-active β-catenin (CA-βCat) in the lateral cortex at E12.5. (**B, D**) Quantification of the fluorescence intensity of Dmrt3 and Dmrta2 staining in electroporated (EP) and non-electroporated (non-EP) cells in the electroporated area shown in (A) and (C). *n* = 40 in each experiment. Dotted lines and circles in (A) and (C) indicate the ventricular surface of the telencephalon and a subset of electroporated cells in the VZ. Scale bar, 50 µm.

The requirement of a Dmrta2 gradient for the development of medial structures raised the question of what regulates the expression of Dmrta2. The canonical Wnt signaling pathway is one possible candidate for this role, given its involvement in the maintenance of neural progenitors in the medial cortex [5]. To assess the possible role of Wnt signaling in Dmrta2 expression, we examined the overexpression of a dominant-negative form of Tcf3 (DN-Tcf3), which lacks the β-catenin-interacting domain and thus inhibits Tcf/Lef-dependent transcription. Here, we also examined the expression of Dmrt3 to investigate whether its expression is under the control of the Wnt signaling pathway, given that it shares a similar expression profile with Dmrta2 during brain development. At 24 h after *in utero* electroporation, the percentage of Dmrta2-positive cells of the total EGFP-positive cells did not change significantly {84.5±2.3 (Control) vs. 87.8±5.3 (DN-Tcf3), *n* = 3, *p* = 0.38}. However, their expression in EGFP-positive electroporated cells significantly decreased compared to that of the controls (Figure 7A, B). Furthermore, the overexpression of a constitutive-active form of β-catenin, which lacks the N-terminal phosphorylation domain essential for its degradation, elevated the expression of Dmrt3 and Dmrta2 in the lateral region of the ventricular zone (Figure 7C, D). Thus, the expression of Dmrt3 and Dmrta2 in the medial cortex, including the cortical hem, is at least partly regulated through canonical Wnt signaling.

## Discussion

Here, we report a critical role for the DM domain-containing protein Dmrta2 in the early embryonic development of the telencephalon. *Dmrta2* mutants showed a dramatic reduction in the size of the dorsal telencephalon and a reduction in the total number of Sox2-positive neural progenitors. These defects suggest malfunctions of neural progenitors in *Dmrta2* mutant telencephalon, which might include slow cell cycle progression, precocious neurogenesis, or increased apoptosis. However, the number of TUNEL-positive cells and cell cycle exit rate did not change significantly during the period of the phenotypic emergence in *Dmrta2* mutants, suggesting that the latter two possibilities are unlikely. In contrast, the cell cycle kinetics of the neural progenitors in *Dmrta2* mutants appeared to change, suggesting aberrant cell cycle progression. Thus, the regulation of cell cycle progression is most likely a predominant function of Dmrta2 in the neural progenitors.

As illustrated by the complete loss of the hippocampus and an extreme shrinkage of the cortical hem, the medial structure defects in *Dmrta2*-mutant brains were considerably more severe than those in the dorsolateral telencephalon. These malformations might be explained by the loss of the cortical hem, which acts as a signaling center by secreting Wnts [32]. The contribution of Dmrta2 to corticogenesis thus appears to depend on medio-lateral patterning of the cortex. Furthermore, in the absence of Dmrta2 function, the medio-lateral gradient of Pax6, which is a key patterning factor in cerebral cortical development, was lost in the dorsal cortex. These observations raise the possibility that Dmrta2 is involved in the patterning during telencephalon development. In this study, Wnt signaling was shown to partly control Dmrta2 expression, suggesting that Dmrta2 functions as a downstream of Wnts in coupling of cell proliferation with cortical patterning. Further studies on the functional relationship between Dmrta2 and the other patterning factors might help to reveal a transcriptional network that will tightly link cell proliferation and patterning in the neural progenitor.

The Wnt-dependent expression of Dmrt3 and Dmrta2 is partly consistent with the results of a recent report showing that a Tcf-binding site in a *Dmrt3* enhancer element is necessary for the expression of *Dmrt3*, although *Dmrta2* expression was not shown to have a clear graded expression in the telencephalon [33]. Our *in situ* hybridization data showed that Dmrta2 is expressed in a medial-high to lateral-low gradient during cortical development, although its gradient is less prominent than that of *Dmrt3*. However, expression of both proteins clearly showed very similar expression pattern of medial-high to lateral-low gradients during cortical development, suggesting that the expression of these two factors are regulated by same signaling pathway. In addition, we observed the residual uniform expression of both Dmrt3 and Dmrta2 in the *Emx1/2* double mutant brains, in which the formation of the cortical hem and Wnt signaling are severely disorganized [23] (D.K. and F.M., unpublished data). These data suggest that the expression of Dmrt3 and Dmrta2 might be regulated by the combination of Wnt-dependent and Wnt-independent pathways.

The vertebrate *Dmrt* gene family proteins were first identified by their homology with *Drosophila melanogaster* Dsx (Double sex) and *Caenorhabditis elegans* mab-3 (male abnormal-3) [34]–[36]. Of the seven mammalian Dmrt proteins, three (Dmrt3, Dmrta1, and Dmrta2) share a highly conserved protein domain of unknown function, called the DMA domain (Dmrta subfamily). Previous reports describe that the Dmrta gene subfamily functions in the nervous system of vertebrates and invertebrates [37]–[40]. In particular, the Dmrta proteins promote neurogenesis by regulating the expression of bHLH transcription factors in *Xenopus* and *Zebrafish* CNS development [37], [40]. However, we observed no significant increases in the expression of Neurogenin2, a proneural factor that promotes neurogenesis, nor did we identify any changes in the neurogenic properties of *Dmrta2* mutants (Figure 4, data not shown). This discrepancy might be explained by the different roles of Dmrt proteins in different biological contexts, and/or by the redundant roles of Dmrt with other proteins.

It is unclear if Dmrta2 maintains the cerebral cortical neural progenitors in a cell-autonomous manner. In this study, we could not address whether Dmrta2 is directly involved in the maintenance of the neocortical progenitors beyond its indirect contributions to cortical hem formation. However, the defects in *Dmrta2* mutant brains appeared to be much more severe in terms of size and morphology than those observed in the cortical hem-ablated mutants generated through the conditional expression of diphtheria toxin-A in the Wnt3a-expressing cells [14], suggesting that the Dmrta2 mutant phenotype reflects the combination of cell-autonomous and non-cell-autonomous Dmrta2 functions in the maintenance of neocortical neural progenitors. Additional work focusing on the cortical hem specific ablation of *Dmrta2* might help to address this question.

## Supporting Information

Figure S1 **Generation of specific antibodies for Dmrt3 and Dmrta2.** Western blotting of the cell lysate from HEK293 cells that were transfected with the expression plasmid for FLAG-Dmrt3, FLAG-Dmrta1, FLAG-Dmrta2, or FLAG-Dmrt7.
(TIF)

Figure S2 **Generation of a conditional mutant for** ***Dmrta2*** **.** (**A**) Schematic representation of the strategy used to generate a *Dmrta2* conditional mutant allele. Exon 2 of the *Dmrta2* gene, which includes a sequence encoding the DM domain of Dmrta2, is flanked by loxP sites. The neo-cassette flanked by FRT sequences was removed by crossing *Dmrta2* mutants with the *ACTB-FLPe* transgenic mice. The *Dmrta2* null and conditional mutants were generated by crossing *Dmrta2^flox/flox^* to the *EIIa-Cre* and the *Nestin-Cre* transgenic mice, respectively. (**B**) Genotyping of *Dmrta2* allele by PCR using the primer sets indicated with blue arrows in (A).
(TIF)

Figure S3 **Cortical hem formation and Cajal-Retzius cell production are severely disorganized in** ***Dmrta2^−/−^*** **embryos. (A)** Gross morphology of embryos and brains from E15.5 *Dmrta2* mutant embryos. **(B, C)** Immunostaining for Otx2 and Prox1 with DAPI staining in *Dmrta2* mutants. Images in (C) show higher magnifications of the boxed area in (B). **(D)** Double immunofluorescence for Tbr1 and Pou3f2. **(E)** Immunofluorescence for Reelin. Dashed lines in (B–D) indicate the ventricular surface. Scale bar, 100 µm.
(TIF)

Table S1 **Microarray analysis of the developing mouse telencephalon.** The expression values of twenty genes that are highly expressed in the E10.5 brains and *Sox2*.
(XLS)
