# Supplementary figures and images for: The Mammalian DM Domain Transcription Factor Dmrta2 Is Required for Early Embryonic Development of the Cerebral Cortex
Source: PLoS One. 2012 Oct 2;7(10):e46577. doi: 10.1371/journal.pone.0046577 (PMC3462758; doi:10.1371/journal.pone.0046577)

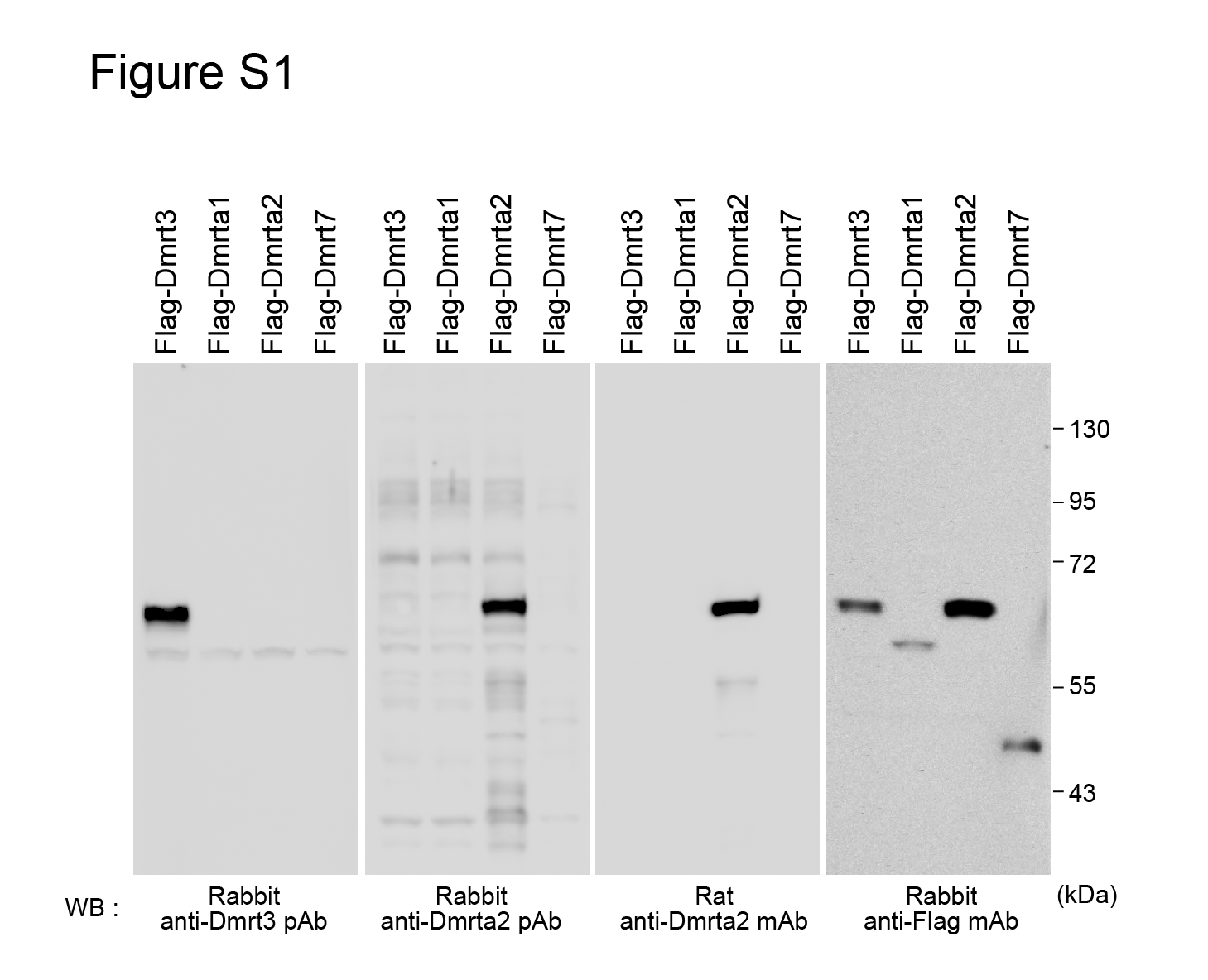

Supplement: Figure S1 — Generation of specific antibodies for Dmrt3 and Dmrta2. Western blotting of the cell lysate from HEK293 cells that were transfected with the expression plasmid for FLAG-Dmrt3, FLAG-Dmrta1, FLAG-Dmrta2, or FLAG-Dmrt7. (TIF) [file pone.0046577.s001.tif]

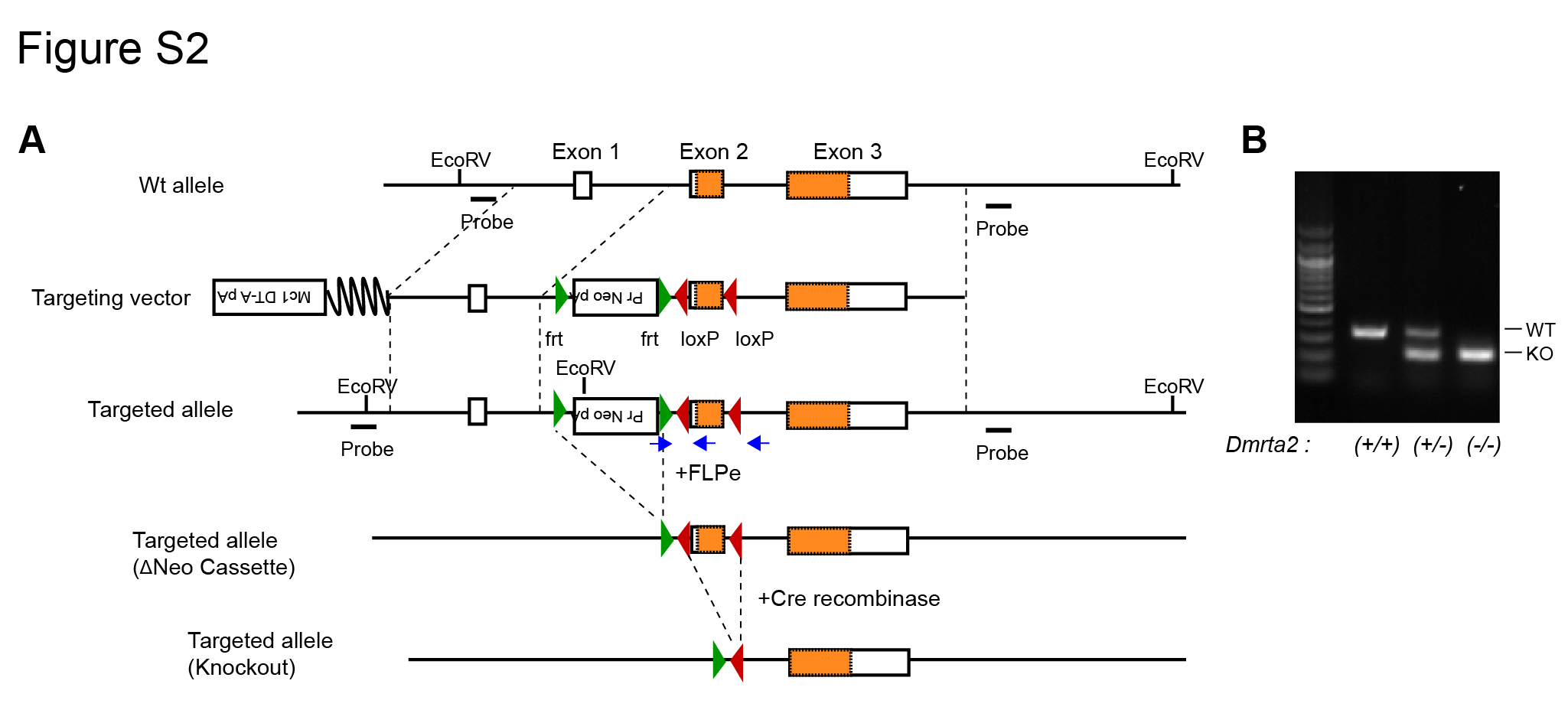

Supplement: Figure S2 — Generation of a conditional mutant for Dmrta2 . (A) Schematic representation of the strategy used to generate a Dmrta2 conditional mutant allele. Exon 2 of the Dmrta2 gene, which includes a sequence encoding the DM domain of Dmrta2, is flanked by loxP sites. The neo-cassette flanked by FRT sequences was removed by crossing Dmrta2 mutants with the ACTB-FLPe transgenic mice. The Dmrta2 null and conditional mutants were generated by crossing Dmrta2flox/flox to the EIIa-Cre and the Nestin-Cre transgenic mice, respectively. (B) Genotyping of Dmrta2 allele by PCR using the primer sets indicated with blue arrows in (A). (TIF) [file pone.0046577.s002.tif]

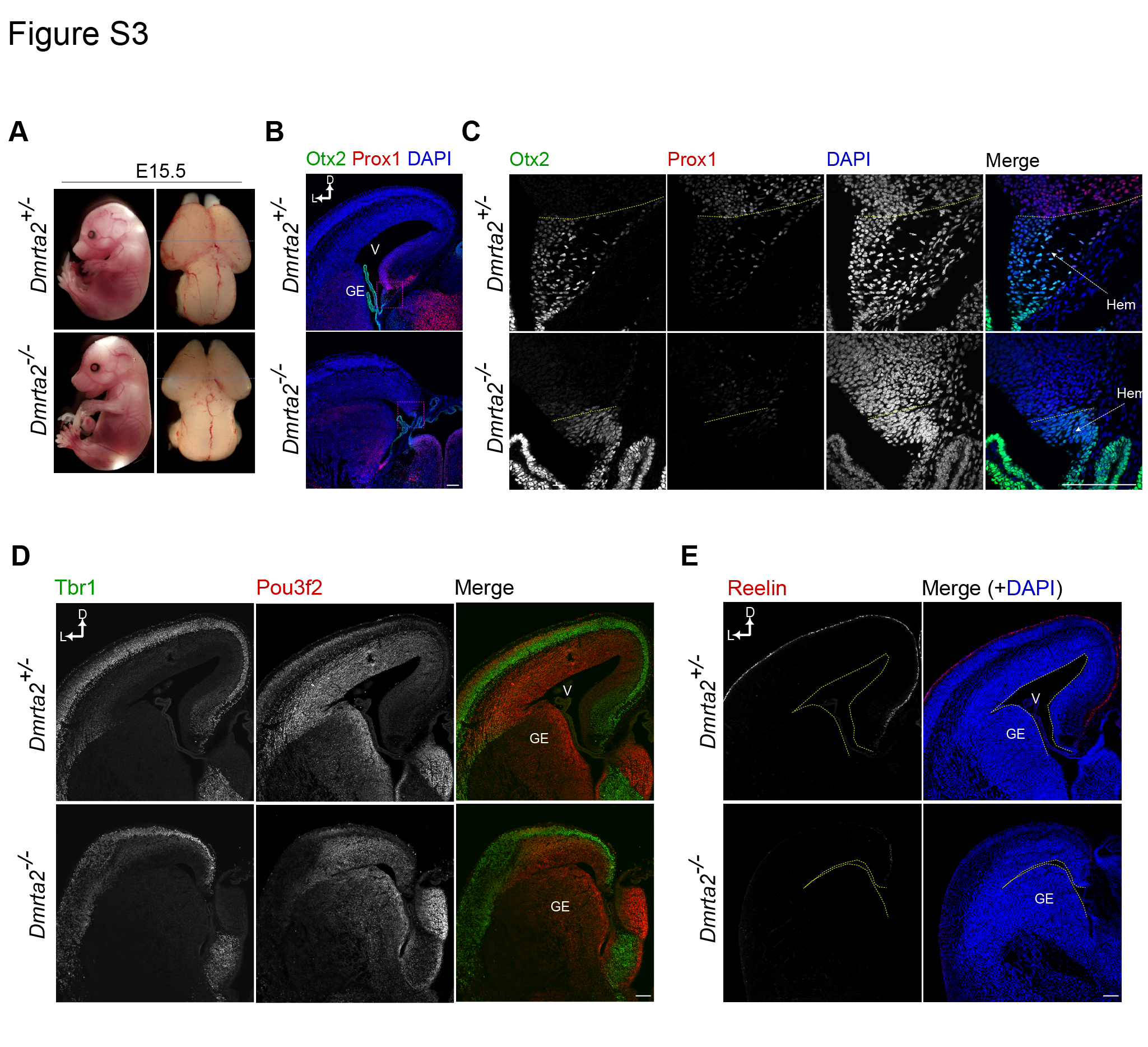

Supplement: Figure S3 — Cortical hem formation and Cajal-Retzius cell production are severely disorganized in Dmrta2−/− embryos. (A) Gross morphology of embryos and brains from E15.5 Dmrta2 mutant embryos. (B, C) Immunostaining for Otx2 and Prox1 with DAPI staining in Dmrta2 mutants. Images in (C) show higher magnifications of the boxed area in (B). (D) Double immunofluorescence for Tbr1 and Pou3f2. (E) Immunofluorescence for Reelin. Dashed lines in (B–D) indicate the ventricular surface. Scale bar, 100 µm. (TIF) [file pone.0046577.s003.tif]
